# Development and validation of the Vietnamese primary care assessment tool

**DOI:** 10.1371/journal.pone.0191181

**Published:** 2018-01-11

**Authors:** Nguyen Thi Hoa, Nguyen Minh Tam, Wim Peersman, Anselme Derese, Jeffrey F. Markuns

**Affiliations:** 1 Department of Family Medicine, Hue University of Medicine and Pharmacy, Hue University, Hue, Vietnam; 2 Department of Family Medicine and Primary Healthcare, Ghent University, Ghent, Belgium; 3 Social and Community Work, Odisee University College, Brussels, Belgium; 4 Global Health Collaborative, Department of Family Medicine, Boston University, Boston, Massachusetts, United States of America; Universite de Bretagne Occidentale, FRANCE

## Abstract

**Objective:**

To adapt the consumer version of the Primary Care Assessment Tool (PCAT) for Vietnam and determine its internal consistency and validity.

**Design:**

A quantitative cross sectional study.

**Setting:**

56 communes in 3 representative provinces of central Vietnam.

**Participants:**

Total of 3289 people who used health care services at health facility at least once over the past two years.

**Results:**

The Vietnamese adult expanded consumer version of the PCAT (VN PCAT-AE) is an instrument for evaluation of primary care in Vietnam with 70 items comprising six scales representing four core primary care domains, and three additional scales representing three derivative domains. Sixteen other items from the original tool were not included in the final instrument, due to problems with missing values, floor or ceiling effects, and item-total correlations. All the retained scales have a Cronbach’s alpha above 0.70 except for the subscale of Family Centeredness.

**Conclusions:**

The VN PCAT-AE demonstrates adequate internal consistency and validity to be used as an effective tool for measuring the quality of primary care in Vietnam from the consumer perspective. Additional work in the future to optimize valid measurement in all domains consistent with the original version of the tool may be helpful as the primary care system in Vietnam further develops.

## Introduction

Quality primary care is an essential component of strong health care systems with good health outcomes [1]. In 1978 at Alma Ata, the World Health Organization (WHO) promoted “primary care” as essential for all health systems. Research from industrialized countries has shown that stronger primary care systems are associated with lower costs and better population health outcomes [1–5]. Studies in the United States and in low- and middle-income countries have also suggested that greater primary care availability is correlated with improved health and a decrease in utilization of high cost health services [6–8]. In 2008, the World Health Organization reiterated their call for all countries to strengthen primary care systems and use primary care as a model to provide care that is equitable and efficient [9, 10].

Primary care in Vietnam is mainly provided by a network of more than 11,000 commune health care centers that provide basic and essential health services to people in every commune. A commune health center (CHC) is usually staffed with a general doctor and some ancillary staff such as a midwife, nurse, assistant doctor of traditional medicine or pharmacist. This network is supplemented by additional outpatient “polyclinics” (staffed by multiple primary care and subspecialist physicians) and district hospitals. People with public health insurance may seek health care services at their registered primary health facility, normally their local commune health center, and can then be referred to a higher level if needed such as district, provincial or central hospitals. Although those with public health insurance generally have free or low-cost access to primary care services through the CHCs, many people believe the quality to be poor and so bypass their CHC at the grassroots level and instead choose to self-pay for services directly at private clinics or hospitals. This pattern of care-seeking behavior has led to serious overcrowding in most upper level referral hospitals, despite potential compromises in quality due to extensive waiting times and short consultations under extreme time pressure. As a result, Vietnam has begun a variety of interventions since 2013 to improve the primary care system [11, 12]. Correspondingly, there is now a great need for valid tools to measure the quality of primary care and assist in evaluating these interventions and their effectiveness.

There are a variety of tools for measuring elements of primary care, however, the Primary Care Assessment Tool (PCAT) developed by Barbara Starfield at the Johns Hopkins Primary Care Policy Center focuses on the core principles of primary care and is one of the few tools designed to assess both structural and process features of primary care [13, 14]. The PCAT family of instruments includes four surveys: the adult consumer-client survey (PCAT-AE), the child consumer-client survey (PCAT-CE), a provider survey and a facility survey. The PCAT-AE is designed to collect information from consumers regarding their experience using health care resources, and it may be used to survey target populations [14].

The PCAT gauges the organizational resources and processes of grassroots health care by evaluating four essential features or core domains of primary care: first contact care (access), longitudinality (continuity), comprehensiveness and coordination. Three other derivative domains are also included in the PCAT: family-centered care, community-oriented care and culturally competent care [15]. Each domain is represented by one or two small scales. Six scales represent the four core domains of primary care: first contact, longitudinal care, coordination of services (coordination domain), comprehensive services available and comprehensive services provided (comprehensiveness domain). Three additional scales represent the three ancillary domains of family centeredness, community orientation and cultural competence. Thus, the original PCAT-AE consists of nine scales representing seven domains [14].

The PCAT-AE has been used and validated in multiple countries and is perhaps one of the most widely studied and applied tools for measuring quality of primary care across the globe [16–19]. Given the proven utility of the tool worldwide, we presumed it to be a useful tool to gauge the quality of primary care as an emerging component of the healthcare system in Vietnam. Although the PCAT-AE has been validated in a variety of countries, specificities of local health systems and patients’ cultural understanding of key concepts may make some elements of the tool less useful or valid. In this study, we developed the Vietnamese Primary Care Assessment Tool based on the consumer-client version of the adult expanded PCAT (VN PCAT-AE) and examined its internal consistency and validity.

## Method

### Translation and adaptation of the PCAT for Vietnam

**Fig 1:**
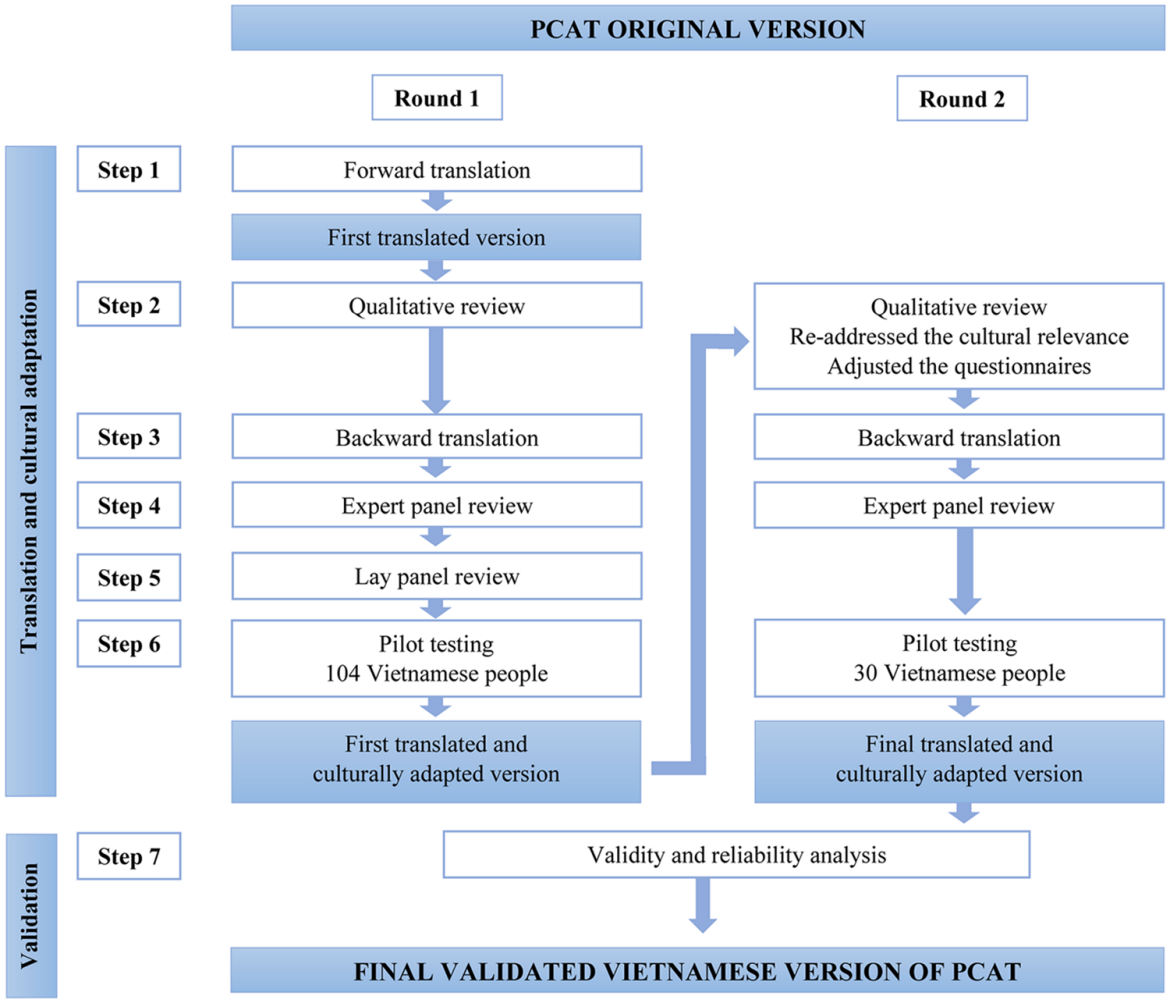
PCAT translation and validation process.

A toolkit developed by the Johns Hopkins Primary Care Policy Center for use of the PCAT in international settings contains a set of recommended steps for valid linguistic and cultural translation of the tool (available upon request from the Center). In our initial adaptation of the tool for Vietnam, all of the recommended translation steps were successfully performed at least once as part of the translation process as shown in the Fig 1. Details of the process used are as follows:

- **Step 1: Forward translation** performed by a bilingual physician and a PhD student whose native tongue was Vietnamese, with experience translating documents from Vietnamese to English, and who was also familiar with use of the PCAT. Translation prioritized preserving the intent over the literal meaning of the items.
- **Step 2: Qualitative review** of the translated survey completed by a group of doctors and researchers from Hanoi Medical School in a focus group discussion; every translated item was reviewed to ensure its clarity, use of common language and conceptual adequacy.
- **Step 3: Backward translation** completed by a woman whose native language is American English and has lived in the US long enough to know the language and routines of daily life but was not already familiar with the specific wording of the original PCAT terms.
- **Step 4: Doctors and health experts in Vietnam and translators jointly reviewed** the forward and backward translations to assess items that were not effectively translated and those which were confusing or generated concerns. A few modifications were made and a consensus translation was produced that was determined appropriate for use in Vietnam.
- **Step 5: Lay panel review** occurred by two different panels of non-subjects (consumers and physicians) to review the translation, identify troublesome items, and propose alternatives.
- **Step 6: Pilot testing** was implemented using a final translated version. The translated version was administered to 104 representative patients who were native Vietnamese speakers and representative in terms of age, gender, and socioeconomic status. Basic descriptive analyses were conducted to ensure adequate distribution of responses. Respondents were debriefed to identify any wording or comprehension problems.

Based on challenges experienced in early efforts to utilize the tool, some important steps were repeated to improve and ensure the high quality of the questionnaire including another qualitative review to re-address the cultural relevance of each item. The research team produced a list of problematic items and proposed solutions, with subsequent backward translation. An expert panel, including family medicine leaders from all medical universities in Vietnam with the specialty of family medicine, reviewed the suitability of each item as well as the words used in the questionnaire, resulting in an updated translation of the questionnaire.

An additional pilot study was then conducted with 30 people living in two communes, and some words and cultural references in specific items were identified for further revision. A final revision was done by the research team after review of all the items and obtaining additional advice from international experts with experience in PCAT validation. The final translated version of the questionnaire for this study was then produced.

The most contentious issue throughout the process was what term to use in place of “primary care provider (PCP)” as this is a completely unknown term in the Vietnamese context. Efforts to address this also impacted the decision to repeat some translation and validation steps. Ultimately through the lay and expert review processes, the term “general doctor” was chosen to most closely represent this concept. Additional substantive changes were to replace or reword items that are not typically present in Vietnam with those that were more contextually relevant. For instance, descriptions of the types of facilities in the affiliation section were changed to use more appropriate terminology relevant to Vietnam. Similarly, some clinical services in the comprehensiveness domain were replaced to ensure items were sufficiently relevant to the Vietnamese context, similar to changes in PCAT versions from other countries[20]. Different country versions of the PCAT often have varying numbers of items to assess this domain, and so two items in the Comprehensiveness (services available) domain (G21 and G22) were completely eliminated and not replaced in the final expert review round due to consensus on the extreme scarcity of the services. Table 1 shows the original and translated items for the items that were most substantially modified.

**Table 1:** Changes in the final translated questionnaires from the original PCAT.

Item code	Original question	Final translated question
	**A. EXTENT OF AFFILIATION WITH A PLACE/DOCTOR**	
A5	What kind of office is your PCP? A hospital emergency room A clinic at a hospital A particular doctor’s office outside a hospital A particular doctor’s office inside a hospital A group office A neighborhood health clinic A work or school clinic	What kind of office is your GENERAL DOCTOR? A commune health center A ward health center An outpatient department of a district hospital An outpatient department of a provincial hospital An outpatient department of center hospital A private clinic of a doctor outside of a hospital A private clinic of a group doctors outside of a hospital Another type of place (Please specify) Not sure/don’t remember
	**G. COMPREHENSIVENESS (SERVICES AVAILABLE)**	
G3	Checking to see if your family is eligible for any social service programs or benefits	Checking to see if your family is eligible for any social service programs or benefits such as: economic, medical, food supports
G9	Tests for lead poisoning	Counseling and treatment for alcoholism
G14	Allergy shots	Allergy treatment
G15	Splinting for a sprained ankle	Temporary fix for broken bone
G16	Removal of wart	Gastric catheter insertion/ nasogastric tube
G24	Suggestions for nursing homecare for someone in your family	Postpartum care of umbilical cord
G25	WIC services (supplemental milk and food program)	Monitoring of a normal pregnancy
	**H. COMPREHENSIVENESS (SERVICES PROVIDED)**	
H2	Home safety, like getting and checking smoke detectors and storing medicines safely	Home safety, like preventing accidents, burning, electric shock and storing medicines safely…
H3	Advice on seat-belt use or child safety seats	Advice on helmet use or safety seats
H9	Ask if you have a gun, its storage or its security	Advice on storing labour equipment safely
H11	How to prevent falls	How to prevent falls for the elderly
	**J. COMMUNITY ORIENTATION**	
J18	Ask family members to be on the Board of Directors or advisory committee?	Collect feedback from patients on health staff performance?

Remaining as consistent as possible with the original tool, the translated questionnaire contained 9 scales with 84 questions representing the primary care domains using a 4-point Likert scale response format (1 = definitely not; 2 = probably not; 3 = probably; and 4 = definitely). An additional “don’t know/don’t remember” option was provided for each item. The questionnaire also included demographic questions such as age, gender, and occupation as well as health condition and degree of affiliation with a usual source of care.

Three questions were refined to identify an individual’s usual source of care as a particular person or place and the strength of that affiliation: (1) “Is there a doctor or place that you usually go if you are sick or need advice about your health?” (2) “Is there a doctor or place that knows you best as a person?” and (3) “Is there a doctor or place that is most responsible for your health care?” A person was considered to have a usual source of care if he or she answered affirmatively to any one of these three questions, and no usual source of care if they provided a negative answer to all three questions. An algorithm based on the responses to these three questions was then used to categorize the strength of affiliation with a primary care source. If all three physicians/places were the same, this was considered evidence of a very strong affiliation. If the response to the first question was the same as for either of the other two questions, then that site was used although the affiliation was categorized as less strong. If the response to the first question was different from the other two responses but the other two responses were the same, then the site where both were the same was used as their primary care source and categorized as a weak affiliation. If all three responses were different, then the site identified in the first question was used and categorized as a very weak affiliation. All subsequent questions asked were intended to refer to this specific person or place. For those with no identifiable source of primary care, subsequent questions were asked about the last place that was visited.

### Data collection

To evaluate the feasibility, internal consistency and validity of the Vietnamese Primary Care Assessment Tool (VN PCAT-AE), a quantitative cross-sectional study was conducted. A multistage and purposive sampling approach was used to select the study sites. Three provinces were chosen purposively to capture the diverse characteristics of central Vietnam: Khanh Hoa, Thua Thien Hue and Quang Tri. To obtain a sample representing the diversity of the country, we purposively selected two to four districts from each province, depending on the number of commune health centers with working physicians. In addition, within these constraints, we chose at least one lowland district, one mountainous district and one urban district when possible. Specifically, in Thua Thien Hue, the survey was done with 24 communes in four districts (six communes per district); in Quang Tri, 14 communes in three districts (one district with six communes and two other districts with four communes); and in Khanh Hoa, two districts with a total of 18 communes were selected, for an overall total of 56 communes.

From each commune, 30 households were selected. Half (15) of the households were selected from a list of patients recently treated at the local CHC. The other households were selected from a commune household list. Another 15 from each list were placed on reserve lists for later use in the case of refusals or non-respondents. On the patient list from the CHC, we started with the household of the first person on the examination list of the CHC (i.e. the most recent patient), and then selected every 10^th^ patient who followed (patients 11, 21, 31…) until the intended sample size was reached. Using a similar technique, we selected every tenth household from a separate list of households in the commune.

Each selected household was visited and the head of household surveyed, as well as one other willing adult (≥18 years old) if available during this home visit. Data collection was conducted from January through August of 2014 and questionnaires were administered through in-person interviews. Only participants who had utilized health care services at a health facility at least once over the past two years were surveyed.

Before the interview, participants received a full explanation of the study’s content and purpose and signed a consent form if they agreed to participate. Refusals were rare and so a response rate was not specifically tracked, but surveyors estimated the refusal and non-response rates at less than 5%. If a household refused or could not be reached after three attempts, then another household was chosen at random from the reserve list. Participants were compensated for their time with small gifts of appreciation (worth $2.50 USD) upon completion of the interview.

This study obtained ethical approval from the Scientific Committee of Hue University of Medicine and Pharmacy on 18th March 2014 and IRB review from Boston University (H-31432).

### Data analysis

All collected questionnaires were cleaned and scanned into a computer for storage and convenient review in the future, followed by entry into EpiData by a group of six students working in pairs. Double data entry was used to check for errors in data entry. Data analysis was performed using SPSS software version 23.0.

Subsequent full validation involved several steps. First, individual items were evaluated on several criteria. Items with a high percentage (≥20%) of item non-response or “don’t know/don’t remember” responses, or items with a large floor or ceiling effect (>80% of respondents chose the lowest or highest answering category) were removed. Next, the item-total correlation for the remaining items in each scale was calculated (item-total correlation before review). Items were removed if the item-total correlation was below 0.30 or if Cronbach’s coefficient alpha for that scale improved substantially when the item was removed. Finally, item-discriminant validity was tested: for each item, the item-total correlation (item-total correlation after review) with the hypothesized scale should be substantially higher than the correlation with the other scales. In the second phase, Cronbach’s coefficient alpha was used to examine how well all items measured the same construct (internal consistency). A value of 0.70 is very often seen as a minimum[21].

The recoding progress and calculation for the sum mean score of domains and subdomains of primary care strictly complied with the guideline PCAT manual issued by John Hopkins University in 1998. For calculating the sum mean scores of domains and subdomains, a mean value was assigned to “not sure/don’t remember” answers as well as to missing values.

## Results

### Characteristics of study population

Table 2 describes the characteristics of the 3289 participants with valid questionnaires. For the extent of affiliation with a place or doctor, results suggested that most participants have a strong (35%) or very strong (29%) affiliation with their doctor, while approximately a third report a weak (29%) or very weak (6.1%) affiliation.

**Table 2:** Characteristics of the participants (n = 3289).

Characteristics	n	%
**Gender (n = 3289)**		
Male	1421	43.2
Female	1868	56.8
**Age (years) (n = 3286)**, Mean: 50.1 (SD:16.6)		
18 to 39	1387	42.2
40 to 59	951	28.9
60 and over	948	28.8
**Education (n = 3267)**		
Completed primary school	986	30.2
Completed secondary school	843	25.8
Completed high school	491	15.0
Completed some university/college	290	8.9
Did not complete primary school	572	17.5
Illiterate	85	2.6
**Occupation (n = 3268)**		
Employed full-time	1725	52.9
Employed part-time	509	15.6
Not employed	585	17.9
Retired/in school	441	13.5
**Living area (n = 3289)**		
Urban	1194	36.3
Rural	2095	63.7
**Self-rated health (n = 3286)**		
Excellent	12	0.4
Very good	185	5.6
Good	1454	44.2
Fair	1330	40.5
Poor	305	9.3
**Chronic problem in last year (n = 3284)**		
Yes	422	13.2
No	2769	86.8
**Trouble with healthcare payment (n = 3006)**		
Yes	532	17.7
No	2474	82.3
**Source of healthcare payment**		
Government health insurance	2469	75.3
Private health insurance	90	2.8
Free or discounted by the health facility	941	28.8
Out of pocket	1591	48.6
**Time affiliated with health facility (n = 3285)**		
Less than 6 months	427	13.3
6 months—1 year	335	10.4
1–2 years	642	20.0
3–4 years	500	15.6
5 years or more	1311	40.8
**Reason to choose this health facility (n = 3283)**		
Patient or someone in family chose it	1837	56.3
Patient was assigned to it	1428	43.7
**Extent of Affiliation with a Place/Doctor (n = 3289)**		
Very weak affiliation	202	6.1
Weak affiliation	972	29.6
Strong affiliation	1146	34.8
Very strong affiliation	969	29.5
**Types of health facility (n = 3289)**		
Commune health center	1506	45.8
Polyclinic	215	6.5
District hospital	389	11.8
Provincial hospital	147	4.5
Central hospital	83	2.5
Private clinic	198	6.0
Pharmacy	127	3.9
Other type of health facility	624	19.0

SD: Standard deviation

### Evaluation of the individual items

Evaluation of the individual items shows that fourteen items were problematic (Table 3). Because of a high percentage of “don’t know/don’t remember” or missing answers (≥20%), two items were removed from the domain of First contact—accessibility (C8 and C10), in addition to three items from the domain Comprehensiveness (services available) (G16, G17, G18) and one item (J12) from the domain of Community orientation.

**Table 3:** Item mean (SD), percentage ‘*don’t know*, *don’t remember/ missing*’, floor/ceiling effect, item total correlation before review, item-total correlation after review and range of item correlation with other domains.

	Item	Item mean (SD)	% missing/ % don't know, don't remember	Floor/ ceiling effect	Item total correlation before review	Item total correlation after review	Range of item correlation with other domains (min/max)
	**B. First contact—utilization**						
B1	When you need a regular general checkup, do you go to your GENERAL DOCTOR before going somewhere else?	2.72 (1.42)	0.0/1.8	38.3/51.8	0.84	0.84	0.03/0.22
B2	When you have a new health problem, do you go to your GENERAL DOCTOR before going somewhere else?	3.17 (1.25)	0.0/0.7	22.6/65.3	0.81	0.81	0.02/0.17
B3	When you have to see a specialist, does your GENERAL DOCTOR have to approve or give you a referral?	2.43 (1.39)	0.2/2.4	45.3/38.8	0.79	0.79	0.02/0.27
	**C. First contact—accessibility**						
C1	Is your GENERAL DOCTOR open on Saturday or Sunday?	2.98 (1.31)	0.1/3.6	27.8/57.5	0.60	0.66	-0.02/-0.15
C2	Is your GENERAL DOCTOR open on at least some weekday evenings until 8 PM?	2.83 (1.32)	0.2/6.1	30.0/49.4	0.52	0.59	-0.002/0.09
C3	When your GENERAL DOCTOR is open and you get sick, would someone from there see you the same day? *	3.79 (0.63)	0.2/1.2	3.7/87.2	Not assessed	Not assessed	Not assessed
C4	When your GENERAL DOCTOR is open, can you get advice quickly over the phone if you need it?	1.67 (1.10)	0.2/9.9	69.3/13.4	0.51	0.54	0.09/0.38
C5	When your GENERAL DOCTOR is closed, is there a phone number you can call when you get sick?	2.13 (1.36)	0.2/6.2	56.4/30.1	0.60	0.65	0.09/0.38
C6	When your GENERAL DOCTOR is closed on Saturday and Sunday and you get sick, would someone from there see you the same day?	3.07 (1.20)	0.1/7.6	21.3/54.1	0.60	0.73	0.10/0.32
C7	When your GENERAL DOCTOR is closed and you get sick during the night, would someone from there see you that night?	3.04 (1.18)	0.0/9.2	21/50.7	0.55	0.70	0.11/0.38
C8	Is it easy to get an appointment for a general check-up there? *	2.42 (1.37)	0.1/93.2	45/36.5	Not assessed	Not assessed	Not assessed
C9	Once you get to your GENERAL DOCTOR, do you have to wait more than 30 minutes before you are checked by the doctor or nurse? *	2.71 (1.30)	0.4/4.7	28.5/45.9	0.39	Not assessed	Not assessed
C10	Do you have to wait a long time or talk to too many people to make an appointment with your GENERAL DOCTOR? *	1.76 (1.14)	0.1/92.4	64.3/14.9	Not assessed	Not assessed	Not assessed
C11	Is it difficult for you to get medical care from your GENERAL DOCTOR when you think it is needed? *	3.42 (1.00)	0.3/1.4	9.7/69.9	0.26	Not assessed	Not assessed
C12	When you have to go to your GENERAL DOCTOR, do you have to take off from work or school to go? *	2.18 (1.36)	0.0/0.7	52.2/32.6	0.24	Not assessed	Not assessed
	**D. ONGOING CARE**						
D1	When you go to your GENERAL DOCTOR’s, are you taken care of by the same doctor or nurse each time?	2.41 (1.40)	0.1/1.6	46.4/39.4	0.49	0.50	0.03/0.23
D2	Do you think your GENERAL DOCTOR understands what you say or ask? *	3.81 (0.49)	0.1/0.7	1.3/88.4	Not assessed	Not assessed	Not assessed
D3	Are your questions to your GENERAL DOCTOR answered in ways that you understand? *	3.81 (0.51)	0.1/0.6	1.3/84.3	Not assessed	Not assessed	Not assessed
D4	If you have a question, can you call and talk to the doctor or nurse who knows you best?	2.13 (1.32)	0.3/5.9	53.7/27.3	0.46	0.48	0.13/0.31
D5	Does your GENERAL DOCTOR give you enough time to talk about your worries or problems?	3.42 (0.91)	0.2/1.2	7.6/63.9	0.37	0.37	0.00/0.26
D6	Do you feel comfortable telling your GENERAL DOCTOR about your worries or problems?	3.47 (0.90)	0.0/0.9	7.8/67.8	0.35	0.36	0.01/0.18
D7	Does your GENERAL DOCTOR know you very well as a person, rather than as someone with a medical problem?	2.17 (1.29)	0.1/2.7	49.2/26.7	0.69	0.69	0.10/0.32
D8	Does your GENERAL DOCTOR know who lives with you?	2.27 (1.35)	0.2/4.1	48.5/32.8	0.70	0.72	0.05/0.36
D9	Does your GENERAL DOCTOR know what problems are most important to you?	2.12 (1.22)	0.2/5.8	47.3/21	0.56	0.58	0.05/0.30
D10	Does your GENERAL DOCTOR know your complete medical history?	2.40 (1.24)	0.1/5.3	37.5/27.4	0.61	0.61	0.10/0.31
D11	Does your GENERAL DOCTOR know about your work or employment?	2.76 (1.33)	0.5/2.8	32.2/47.2	0.62	0.62	0.09/0.31
D12	Would your GENERAL DOCTOR know if you had trouble getting or paying for medicines you needed?	1.68 (1.04)	0.3/7.4	63.9/11.4	0.56	0.58	0.10/0.26
D13	Does your GENERAL DOCTOR know about all the medications you are taking?	2.33 (1.24)	0.5/3.8	38.9/27.5	0.51	0.53	0.02/0.34
D14	Could you change your GENERAL DOCTOR if you wanted to?	2.55 (1.34)	0.2/2.7	37.7/39.9	-0.04	Not assessed	Not assessed
D15	Would you change from your GENERAL DOCTOR to somewhere else if it was easy to do?	2.62 (1.33)	0.1/3.1	34.4/42.0	0.25	Not assessed	Not assessed
	**E. COORDINATION**						
E6	Did your GENERAL DOCTOR suggest you go to the specialist or special service? (848)	2.38 (1.47)	0.0/0.1	52.1/43.9	0.75	0.75	0.03/0.35
E7	Did your GENERAL DOCTOR know you made these visits to the specialist or special service? (843)	2.50 (1.40)	0.0/0.7	42.9/42.4	0.76	0.76	0.06/0.32
E8	Did your GENERAL DOCTOR discuss with you different places you could have gone to get help with that problem? (837)	2.50 (1.40)	0.1/1.3	43.2/40.9	0.73	0.73	0.05/0.25
E9	Did your GENERAL DOCTOR or someone working with your GENERAL DOCTOR help you make the appointment for that visit? (799)	1.46 (0.98)	0.1/5.8	74.7/9.7	0.58	0.58	0.05/0.20
E10	Did your GENERAL DOCTOR write down any information for the specialist about the reason for the visit? (824)	2.14 (1.39)	0.1/2.8	55.6/32.0	0.71	0.71	-0.03/0.36
E11	Does your GENERAL DOCTOR know what the results of the visit were? (824)	2.14 (1.33)	0.4/2.6	52.1/28.0	0.65	0.65	0.03/0.23
E12	After you went to the specialist or special service, did your GENERAL DOCTOR talk with you about what happened at the visit? (829)	1.88 (1.26)	0.1/2.5	62.7/22.4	0.63	0.63	0.02/0.30
E13	Does your GENERAL DOCTOR seem interested in the quality of care you get from that specialist or special service? (796)	1.87 (1.20)	0.4/6.6	56.8/16.8	0.65	0.65	0.02/0.37
	**G. COMPREHENSIVENESS (SERVICES AVAILABLE)**						
G1	Answers to questions about nutrition or diet	3.39 (1.10)	0.0/3.3	15.4/72.1	0.34	0.34	0.09/0.25
G2	Immunizations (shots)	3.20 (1.24)	0.1/3.9	22.1/67.3	0.52	0.52	0.06/0.26
G3	Checking to see if your family is eligible for any social service programs or benefits such as: economic, medical, food supports	2.26 (1.34)	0.3/11.3	48.5/31.6	0.4	0.4	0.10/0.30
G4	Dental check up	3.09 (1.28)	0.0/4.3	24.6/61.8	0.62	0.62	0.03/0.16
G5	Treatment by a dentist	2.14 (1.33)	0.1/11.1	53.3/28.6	0.46	0.46	-0.05/-0.22
G6	Family planning or birth control methods	3.25 (1.16)	0.2/8.0	18.4/64.4	0.59	0.59	0.02/0.27
G7	Substance or drug abuse counseling or treatment	2.27 (1.29)	0.1/17.5	45.6/27.2	0.62	0.62	0.09/0.38
G8	Counseling for mental health problems	2.40 (1.30)	0.1/17.0	41.2/30.7	0.65	0.65	0.03/0.37
G9	Counseling and treatment for alcoholism	2.12 (1.27)	0.5/15.4	51/24.3	0.62	0.62	0.10/0.45
G10	Sewing up a cut that needs stitches	3.44 (1.05)	0.4/4.1	13.4/73.1	0.64	0.64	0.01/0.17
G11	Counseling and testing for HIV/AIDS	2.55 (1.31)	0.5/14.8	36.9/36.9	0.61	0.61	0.00/0.28
G12	Ear check up	3.24 (1.20)	0.1/4.7	20.2/66.7	0.65	0.65	0.02/0.14
G13	Eye check up	3.27 (1.18)	0.1/4.3	19.1/67.9	0.64	0.64	0.01/0.15
G14	Allergy treatment	3.23 (1.17)	0.3/11.7	18.1/64.2	0.47	0.47	0.00/0.14
G15	Temporary fix for broken bone	3.27 (1.13)	0.4/6.3	16.4/64.7	0.63	0.63	-0.02/0.20
G16	Gastric catheter insertion/ nasogastric tube*	1.95 (1.25)	0.3/20.0	59/22	Not assessed	Not assessed	Not assessed
G17	PAP tests for cervical cancer*	1.82 (1.18)	0.1/25.9	62.8/17	Not assessed	Not assessed	Not assessed
G18	Rectal exams or sigmoidoscopy exams to test for bowel cancer*	1.76 (1.13)	0.2/27.1	64.2/14.4	Not assessed	Not assessed	Not assessed
G19	Smoking counseling	2.18 (1.29)	0.4/14.3	49.6/25.7	0.57	0.57	0.12/0.46
G20	Prenatal care	3.25 (1.15)	0.4/7.8	18/63.8	0.69	0.69	0.08/0.24
G23	Changes in mental or physical abilities that are normal with getting older	2.63 (1.33)	0.2/9.9	36.2/40.2	0.44	0.44	0.00/0.36
G24	Postpartum care of umbilical cord	3.17 (1.19)	0.0/8.9	19.8/60.4	0.70	0.70	0.10/0.23
G25	Monitoring of a normal Pregnancy	3.34 (1.12)	0.0/7.0	16.4/68.2	0.67	0.67	0.11/0.28
	**H. COMPREHENSIVENESS (SERVICES PROVIDED)**						
H1	Advice about healthy foods and unhealthy foods	3.41 (1.13)	0.1/1.1	16.7/75.1	0.43	0.43	0.07/0.30
H2	Home safety, like preventing accidents, burning, electric shock and storing medicines safely…	2.03 (1.31)	0.0/4.9	58.7/26.1	0.68	0.68	0.11/0.32
H3	Advice on helmet use or safety seats	1.70 (1.18)	0.2/4.7	71.0/17.9	0.67	0.67	0.09/0.33
H4	Ways to handle family conflicts that may arise from time to time	1.51 (1.01)	0.5/5.3	76.7/11	0.64	0.64	0.13/0.33
H5	Advice about appropriate exercise for you	2.82 (1.36)	0.2/3.0	33.1/51.9	0.56	0.56	-0.02/0.27
H6	Tests for cholesterol levels in your blood	2.07 (1.33)	0.3/8.0	57.5/27.3	0.48	0.48	0.00/0.27
H7	Checking on and discussing the medications you are taking	2.76 (1.35)	0.3/2.6	33.9/48.2	0.55	0.55	0.06/0.29
H8	Possible exposures to harmful substances in your home, at work, or in your neighborhood	1.73 (1.14)	0.2/0.2	67.3/15.4	0.69	0.69	0.08/0.37
H9	Advice on storing labour equipmentsafely	1.64 (1.14)	0.2/4.8	73.3/15.9	0.71	0.71	0.10/0.37
H10	How to prevent hot water burns	1.91 (1.28)	0.3/5.0	63.8/23.2	0.74	0.74	0.12/0.32
H11	How to prevent falls for the elderly	2.22 (1.38)	0.5/4.9	53.6/33.2	0.68	0.68	0.09/0.29
	**I. FAMILY-CENTEREDNESS**						
I1	Does your GENERAL DOCTOR ask you about your ideas and opinions when planning treatment and care for you or a family member?	2.30 (1.37)	0.3/2.5	49.3/34.2	0.80	0.80	0.09/0.36
I2	Has your GENERAL DOCTOR asked about illnesses or problems that might run in your family?	2.51 (1.38)	0.3/3.2	41.7/40.8	0.77	0.77	0.10/0.31
I3	Would your GENERAL DOCTOR meet with members of your family if you thought it would be helpful?	2.23 (1.28)	0.3/5.9	47.3/25.6	0.77	0.77	0.09/0.39
	**J. COMMUNITY ORIENTATION**						
J1	Does anyone at your GENERAL DOCTOR’s office ever make home visits?	1.52 (1.07)	0.0/0.8	79.4/13.7	0.59	0.59	0.13/0.43
J2	Does your GENERAL DOCTOR know about the important health problems of your neighbourhood?	2.41 (1.25)	0.1/7.9	38.0/28.4	0.64	0.64	0.11/0.44
J3	Does your GENERAL DOCTOR get opinions and ideas from people that will help to provide better health care?	3.11 (1.12)	0.1/6.1	17.8/50.7	0.74	0.74	0.10/0.37
	Does your GENERAL DOCTOR do any of the following to help determine the effectiveness of his/her services/programs?						
J11	Surveys of patients to see if the services are meeting people’s needs?	2.56 (1.32)	0.0/4.0	37.3/37.5	0.72	0.72	0.01/0.34
J12	Surveys in the community to find out about health problems s/he should know about? *	2.15 (1.23)	0.1/20.0	47.9/21.7	Not assessed	Not assessed	Not assessed
J18	Collect feedback from patients on health staff performance?	2.47 (1.28)	0.1/14.7	37.9/31.7	0.72	0.72	0.06/0.36
	**K. CULTURALLY COMPETENT**						
K1	Would you recommend your GENERAL DOCTOR to a friend or relative?	2.44 (1.35)	0.1/1.4	42.9/35.5	0.84	0.84	0.03/0.26
K2	Would you recommend your GENERAL DOCTOR to someone who does not speak Vietnamese well?	1.91 (1.20)	0.0/6.1	59.4/17.8	0.86	0.86	0.03/0.23
K3	Would you recommend your GENERAL DOCTOR to someone who uses folk medicine, such as herbs or homemade medicines, or has special beliefs about health care?	2.04 (1.24)	0.1/7.2	54.8/20.4	0.85	0.85	0.06/0.18

SD: Standard deviation;

*: Removed from further analysis

Next, items with a large floor or ceiling effect (>80%) were identified, including one item from the domain of First contact—accessibility (C3) and two items from the domain of Ongoing care (D2 and D3). Item-total correlations for the remaining items in each scale were then used to identify those whose item-total correlation was below 0.30 including two items from the domain of First contact—accessibility (C11, C12) and two items from the domain of Ongoing care (D14 and D15). Finally, Cronbach’s alpha was assessed (see Table 4) and improved substantially (from 0.65 to 0.71) for the first contact-access domain when item C9 was removed. For all items, the item-total correlation with the hypothesized scale was higher than the correlation with the other scales (see S1 Table).

**Table 4:** Descriptive statistics of the domains scales.

Domain	Number of items in the original version (Total: 86)	Number of items in the Vietnamese version (Total: 70)	Mean (SD)	Cronbach’s Alpha
First contact—utilization	3	3	2.78 (1.10)	0.74
First contact—access	12	6	2.62 (0.83)	0.71
Ongoing Care	15	11	2.49 (0.68)	0.77
Coordination	8	8	2.12 (0.90)	0.84
Comprehensiveness (Services Available)	25	20	2.91 (0.71)	0.90
Comprehensiveness (Services Provided)	11	11	2.18 (0.77)	0.84
Family-Centeredness	3	3	2.36 (1.05)	0.68
Community Orientation	6	5	2.40 (0.83)	0.71
Culturally Competent	3	3	2.14 (1.08)	0.80

SD: Standard deviation

### Internal consistency of the different scales

Based on these parameters, 70 items of the VN PCAT-AE were determined to be appropriate for use in this population, to represent four core domains with six scales and three derivative domains with three scales (Table 4). Except for the scale of Family Centeredness, all of the retained scales have a Cronbach’s alpha above 0.70.

### Evaluation within subpopulations

The robustness of the results was explored in different subpopulations such as rural and urban populations, provinces, populations from the CHC consumer’s list and from the community household list. The obtained results are highly stable, however there were a few items that showed a poorer fit in some subpopulations: item C2 and item G2 in Quang Tri province, item G1 in Khanh Hoa province and item G23 in the urban population.

## Discussion

Strictly applying standardized guidelines for translation and adaptation followed by a routine psychometric validation method, we confirmed the Vietnamese PCAT (VN PCAT-AE) to be a valid and reliable instrument for the Vietnamese context, making this the first proven tool developed in Vietnam for comprehensive evaluation of primary care.

The VN PCAT-AE successfully measures all of the important domains of primary care with six scales representing four core primary care domains: first contact accessibility and utilization (first contact domain), ongoing care, coordination care, comprehensiveness-services available and comprehensiveness-services provided (comprehensiveness domain). It also successfully measures another three derivative domains of family centeredness, community orientation and cultural competence.

The VN PCAT-AE retains most major characteristics of the original PCAT version with 70 valid items. It is quite similar to PCAT versions in Argentina and South Africa with a few items determined not to be appropriate in these settings and with the addition of questions more relevant to their contexts [20, 22]. In other countries, some researchers have shortened the questionnaires by rearranging items into different scales or the addition or subtraction of scales [16, 17, 19]. We however sought to maintain the integrity of the original tool to the utmost degree possible.

It is also important to note, however, that the total absence or gross inadequacy of services in a specific domain in a certain country or setting is likely to result in psychometric qualities that threaten the validity of the tool in that domain. In Vietnam, despite of a series of great strides and improvements over the last 20 years, the primary care system is still in an early phase of development and many improvements have not yet been widely and systematically implemented throughout the entire country. In particular, a substantial floor effect may be found as a vast majority of patients in this study reported the absence of a variety of services. For instance, many questions related to appointments were removed from the access domain because of the absence of appointment systems in Vietnam, and thus resulted in removing half of the questions from this domain. While the access domain in the VN PCAT-AE remains an overall valid measure of validity, with the removal of so many items related to appointments, it may no longer maintain the same level of integrity in this domain compared with the original tool.

With primary care services in Vietnam improving, however, it is possible that some questions removed from the tool may become more valid in the future as the primary care system becomes more sophisticated and thus future researchers may want to consider reintegrating some of these questions in the tool and reassessing their validity. Recent positive changes in policy and planning by the Ministry of Health and other government entities for family medicine development and strengthening of the primary care network are anticipated to lead to significant system improvements in the future.

This study has several potential limitations. Firstly, the sample was not recruited randomly in an effort to purposively capture the diverse characteristics of the population in the Central region. Secondly, it was a home survey in which the head of household and one additional adult member were surveyed at time of the visit without a systemic method in place for choosing the additional adult member if more than one might be available, and therefore potentially introducing another source of bias.

In spite of these limitations, the Vietnamese PCAT version VN PCAT-AE demonstrates adequate validity and reliability to be used as an effective tool for comprehensively measuring the quality of primary care in Vietnam from the consumer perspective.

## Supporting information

S1 Table Item correlation with domain scores after review (item convergent validity and item discriminant validity). (DOCX)

S1 Dataset Vietnam PCAT consumer data. (SAV)
