# Supplementary material for: Development and validation of the Vietnamese primary care assessment tool
Source: PLoS One. 2018 Jan 11;13(1):e0191181. doi: 10.1371/journal.pone.0191181 (PMC5764365; doi:10.1371/journal.pone.0191181)
Supplement: S1 Table — (DOCX) [file pone.0191181.s001.docx]

**Supporting information**

**S1 Table. Item correlation with domain scores after review (item convergent validity and item discriminant validity)**

|  |  | **First contact – utilization** | **First contact – access** | **Ongoing Care** | **Coordination Care** | **Comprehensiveness (Services Available)** | **Comprehensiveness (Services Provided)** | **Family-Centeredness** | **Community Orientation** | **Culturally Competent** |
| --- | --- | --- | --- | --- | --- | --- | --- | --- | --- | --- |
| **B. First contact – utilization** | |  |  |  |  |  |  |  |  |  |
| B1 | When you need a regular general checkup, do you go to your GENERAL DOCTOR before going somewhere else? | **0.84** | 0.03 | 0.13 | 0.22 | 0.19 | 0.12 | 0.09 | 0.13 | 0.10 |
| B2 | When you have a new health problem, do you go to your GENERAL DOCTOR before going somewhere else? | **0.81** | 0.02 | 0.15 | 0.17 | 0.14 | 0.09 | 0.12 | 0.13 | 0.10 |
| B3 | When you have to see a specialist, does your GENERAL DOCTOR have to approve or give you a referral? | **0.79** | 0.02 | 0.16 | 0.27 | 0.15 | 0.14 | 0.08 | 0.12 | 0.08 |
| **C. First contact – access** | |  |  |  |  |  |  |  |  |  |
| C1 | Is your GENERAL DOCTOR open on Saturday or Sunday? | -0.15 | **0.66** | 0.03 | 0.05 | -0.02 | 0.12 | 0.13 | 0.14 | 0.03 |
| C2 | Is your GENERAL DOCTOR open on at least some weekday evenings until 8 PM? | -0.15 | **0.59** | -0.002 | 0.05 | 0.07 | 0.17 | 0.06 | 0.8 | 0.9 |
| C4 | C4 When your GENERAL DOCTOR is *open*, can you get advice quickly over the phone if you need it? | 0.09 | **0.54** | 0.38 | 0.17 | 0.1 | 0.23 | 0.27 | 0.35 | 0.22 |
| C5 | When your GENERAL DOCTOR is *closed*, is there a phone number you can call when you get sick? | 0.12 | **0.65** | 0.38 | 0.19 | 0.09 | 0.24 | 0.24 | 0.3 | 0.2 |
| C6 | When your GENERAL DOCTOR is *closed* on *Saturday and Sunday* and you get sick, would someone from there see you the same day? | 0.1 | **0.73** | 0.12 | 0.11 | 0.32 | 0.12 | 0.28 | 0.22 | 0.11 |
| C7 | When your GENERAL DOCTOR is *closed* and you get sick *during the night*, would someone from there see you that night? | 0.12 | **0.7** | 0.15 | 0.12 | 0.38 | 0.13 | 0.26 | 0.23 | 0.11 |
| **D. Ongoing Care** | |  |  |  |  |  |  |  |  |  |
| D1 | When you go to your GENERAL DOCTOR’s, are you taken care of by the *same* doctor or nurse each time? | 0.03 | 0.04 | **0.50** | 0.16 | -0.15 | 0.07 | 0.11 | 0.23 | 0.10 |
| D4 | If you have a question, can you call and talk to *the doctor or nurse who knows you best*? | 0.13 | 0.31 | **0.48** | 0.19 | 0.15 | 0.29 | 0.17 | 0.30 | 0.14 |
| D5 | Does your GENERAL DOCTOR give you enough time to talk about your worries or problems? | 0.00 | 0.07 | **0.37** | 0.05 | 0.07 | 0.14 | 0.16 | 0.26 | 0.00 |
| D6 | Do you feel comfortable telling your GENERAL DOCTOR about your worries or problems? | -0.03 | 0.11 | **0.36** | 0.08 | 0.03 | 0.09 | 0.18 | 0.18 | 0.01 |
| D7 | Does your GENERAL DOCTOR know you very well as a *person*, rather than as someone with a medical problem? | 0.20 | 0.16 | **0.69** | 0.21 | 0.10 | 0.22 | 0.20 | 0.32 | 0.13 |
| D8 | Does your GENERAL DOCTOR know who lives with you? | 0.19 | 0.15 | **0.72** | 0.22 | 0.05 | 0.20 | 0.24 | 0.36 | 0.08 |
| D9 | Does your GENERAL DOCTOR know what problems are most important to you? | 0.05 | 0.23 | **0.58** | 0.30 | 0.07 | 0.29 | 0.19 | 0.30 | 0.19 |
| D10 | Does your GENERAL DOCTOR know your complete medical history? | 0.12 | 0.10 | **0.61** | 0.23 | 0.18 | 0.31 | 0.24 | 0.31 | 0.17 |
| D11 | Does your GENERAL DOCTOR know about your work or employment? | 0.18 | 0.11 | **0.62** | 0.17 | 0.15 | 0.20 | 0.23 | 0.31 | 0.09 |
| D12 | Would your GENERAL DOCTOR know if you had trouble getting or paying for medicines you needed? | 0.16 | 0.17 | **0.58** | 0.24 | 0.10 | 0.24 | 0.17 | 0.26 | 0.25 |
| D13 | Does your GENERAL DOCTOR know about all the medications you are taking? | 0.02 | 0.14 | **0.53** | 0.27 | 0.13 | 0.31 | 0.25 | 0.34 | 0.15 |
| **E. COORDINATION** | |  |  |  |  |  |  |  |  |  |
| E6 | Did your GENERAL DOCTOR suggest you go to the specialist or special service? (848) | 0.35 | 0.08 | 0.19 | **0.75** | 0.06 | 0.10 | 0.12 | 0.12 | 0.03 |
| E7 | Did your GENERAL DOCTOR know you made these visits to the specialist or special service? (843) | 0.32 | 0.08 | 0.25 | **0.76** | 0.06 | 0.10 | 0.16 | 0.13 | 0.09 |
| E8 | Did your GENERAL DOCTOR discuss with you different places you could have gone to get help with that problem? (837) | 0.20 | 0.14 | 0.25 | **0.73** | 0.05 | 0.21 | 0.20 | 0.22 | 0.08 |
| E9 | Did your GENERAL DOCTOR or someone working with your GENERAL DOCTOR help you make the appointment for that visit? (799) | 0.07 | 0.12 | 0.20 | **0.57** | 0.10 | 0.16 | 0.14 | 0.14 | 0.05 |
| E10 | Did your GENERAL DOCTOR write down any information for the specialist about the reason for the visit? (824) | 0.36 | 0.09 | 0.16 | **0.71** | 0.06 | 0.08 | 0.07 | 0.07 | -0.03 |
| E11 | Does your GENERAL DOCTOR know what the results of the visit were? (824) | 0.03 | 0.13 | 0.23 | **0.65** | 0.04 | 0.18 | 0.23 | 0.20 | 0.14 |
| E12 | After you went to the specialist or special service, did your GENERAL DOCTOR talk with you about what happened at the visit? (829) | 0.02 | 0.20 | 0.30 | **0.63** | 0.11 | 0.29 | 0.26 | 0.29 | 0.07 |
| E13 | Does your GENERAL DOCTOR seem interested in the quality of care you get from that specialist or special service? (796) | 0.02 | 0.19 | 0.37 | **0.65** | 0.09 | 0.37 | 0.28 | 0.32 | 0.10 |
| **G. Comprehensiveness (Services Available)** | |  |  |  |  |  |  |  |  |  |
| G1 | Answers to questions about nutrition or diet | 0.09 | 0.19 | 0.21 | 0.09 | **0.34** | 0.25 | 0.24 | 0.25 | 0.15 |
| G2 | Immunizations(shots) | 0.26 | 0.13 | 0.18 | 0.06 | **0.52** | 0.22 | 0.17 | 0.21 | 0.09 |
| G3 | Checking to see if your family is eligible for any social service programs or benefits such as: economic, medical, food supports | 0.22 | 0.10 | 0.30 | 0.16 | **0.40** | 0.20 | 0.18 | 0.22 | 0.13 |
| G4 | Dental check up | 0.09 | 0.07 | -0.06 | -0.03 | **0.62** | 0.16 | 0.15 | 0.05 | 0.03 |
| G5 | Treatment by a dentist | -0.05 | 0.07 | -0.22 | -0.10 | **0.46** | 0.12 | 0.07 | -0.09 | 0.05 |
| G6 | Family planning or birth control methods | 0.27 | 0.15 | 0.14 | 0.02 | **0.59** | 0.24 | 0.17 | 0.21 | 0.08 |
| G7 | Substance or drug abuse counseling or treatment | 0.13 | 0.25 | 0.22 | 0.16 | **0.62** | 0.38 | 0.26 | 0.34 | 0.09 |
| G8 | Counseling for mental health problems | 0.03 | 0.21 | 0.13 | 0.14 | **0.65** | 0.37 | 0.24 | 0.29 | 0.07 |
| G9 | Counseling and treatment for alcoholism | 0.16 | 0.24 | 0.24 | 0.20 | **0.62** | 0.45 | 0.19 | 0.32 | 0.10 |
| G10 | Sewing up a cut that needs stitches | 0.16 | 0.10 | -0.04 | -0.02 | **0.64** | 0.16 | 0.17 | 0.09 | 0.01 |
| G11 | Counseling and testing for HIV/AIDS | 0.00 | 0.15 | 0.04 | 0.05 | **0.61** | 0.28 | 0.22 | 0.18 | 0.02 |
| G12 | Ear check up | 0.06 | 0.04 | -0.07 | -0.02 | **0.65** | 0.14 | 0.12 | 0.03 | 0.02 |
| G13 | Eye check up | 0.06 | 0.05 | -0.05 | -0.01 | **0.64** | 0.15 | 0.14 | 0.04 | 0.01 |
| G14 | Allergy treatment | 0.00 | 0.09 | 0.00 | 0.02 | **0.47** | 0.14 | 0.12 | 0.13 | 0.08 |
| G15 | Temporary fix for broken bone | 0.20 | 0.12 | -0.05 | -0.02 | **0.63** | 0.14 | 0.17 | 0.08 | 0.02 |
| G19 | Smoking counseling | 0.12 | 0.24 | 0.23 | 0.14 | **0.57** | 0.46 | 0.17 | 0.32 | 0.12 |
| G20 | Prenatal care | 0.22 | 0.15 | 0.08 | 0.09 | **0.69** | 0.24 | 0.20 | 0.14 | 0.16 |
| G23 | Changes in mental or physical abilities that are normal with getting older | 0.00 | 0.13 | 0.20 | 0.14 | **0.44** | 0.36 | 0.29 | 0.31 | 0.21 |
| G24 | Postpartum care of umbilical cord | 0.18 | 0.16 | 0.11 | 0.10 | **0.70** | 0.23 | 0.23 | 0.17 | 0.14 |
| G25 | Monitoring of a normal pregnancy | 0.28 | 0.13 | 0.13 | 0.11 | **0.67** | 0.25 | 0.21 | 0.19 | 0.12 |
| **H. Comprehensiveness (Services Provided)** | |  |  |  |  |  |  |  |  |  |
| H1 | Advice about healthy foods and unhealthy foods | 0.07 | 0.18 | 0.27 | 0.12 | 0.22 | **0.43** | 0.27 | 0.30 | 0.14 |
| H2 | Home safety, like preventing accidents, burning, electric shock and storing medicines safely… | 0.11 | 0.11 | 0.32 | 0.16 | 0.24 | **0.68** | 0.20 | 0.31 | 0.11 |
| H3 | Advice on helmet use or safety seats | 0.12 | 0.15 | 0.32 | 0.21 | 0.27 | **0.67** | 0.17 | 0.33 | 0.09 |
| H4 | Ways to handle family conflicts that may arise from time to time | 0.13 | 0.20 | 0.32 | 0.24 | 0.22 | **0.64** | 0.20 | 0.33 | 0.15 |
| H5 | Advice about appropriate exercise for you | -0.02 | 0.09 | 0.14 | 0.12 | 0.27 | **0.56** | 0.18 | 0.23 | 0.10 |
| H6 | Tests for cholesterol levels in your blood | 0.06 | 0.09 | 0.00 | 0.00 | 0.27 | **0.48** | 0.11 | 0.09 | 0.07 |
| H7 | Checking on and discussing the medications you are taking | 0.06 | 0.14 | 0.23 | 0.19 | 0.21 | **0.55** | 0.20 | 0.29 | 0.09 |
| H8 | Possible exposures to harmful substances in your home, at work, or in your neighborhood | 0.14 | 0.20 | 0.31 | 0.28 | 0.31 | **0.69** | 0.23 | 0.37 | 0.08 |
| H9 | Advice on storing labor equipment’s safely | 0.10 | 0.19 | 0.30 | 0.21 | 0.27 | **0.71** | 0.20 | 0.37 | 0.13 |
| H10 | How to prevent hot water burns | 0.12 | 0.21 | 0.26 | 0.15 | 0.29 | **0.74** | 0.17 | 0.32 | 0.15 |
| H11 | How to prevent falls for the elderly | 0.09 | 0.19 | 0.22 | 0.15 | 0.29 | **0.68** | 0.19 | 0.29 | 0.17 |
| **I. Family-Centeredness** | |  |  |  |  |  |  |  |  |  |
| I1 | Does your GENERAL DOCTOR ask you about *your* ideas and opinions when planning treatment and care for you or a family member? | 0.09 | 0.21 | 0.29 | 0.24 | 0.23 | 0.25 | **0.80** | 0.36 | 0.20 |
| I2 | Has your GENERAL DOCTOR asked about illnesses or problems that might run in your family? | 0.10 | 0.24 | 0.23 | 0.16 | 0.24 | 0.21 | **0.77** | 0.31 | 0.24 |
| I3 | Would your GENERAL DOCTOR meet with members of your family if you thought it would be helpful? | 0.09 | 0.30 | 0.31 | 0.18 | 0.24 | 0.25 | **0.77** | 0.39 | 0.19 |
| **J. Community Orientation** | |  |  |  |  |  |  |  |  |  |
| J1 | Does anyone at your GENERAL DOCTOR’s office ever make home visits? | 0.13 | 0.25 | 0.43 | 0.29 | 0.16 | 0.28 | 0.31 | **0.59** | 0.14 |
| J2 | Does your GENERAL DOCTOR know about the important health problems of your neighborhood? | 0.23 | 0.27 | 0.44 | 0.16 | 0.22 | 0.31 | 0.28 | **0.64** | 0.11 |
| J3 | Does your GENERAL DOCTOR get opinions and ideas from people that will help to provide better health care? | 0.1 | 0.19 | 0.35 | 0.15 | 0.19 | 0.33 | 0.37 | **0.74** | 0.13 |
|  | Does your GENERAL DOCTOR do any of the following to help determine the effectiveness of his/her services/programs? |  |  |  |  |  |  |  |  |  |
| J11 | Surveys of patients to see if the services are meeting people’s needs? | 0.01 | 0.22 | 0.33 | 0.16 | 0.15 | 0.34 | 0.27 | **0.72** | 0.14 |
| J18 | Collect feedback from patients on health staff performance? | 0.06 | 0.21 | 0.22 | 0.11 | 0.29 | 0.36 | 0.32 | **0.72** | 0.15 |
| **K. Culturally Competent** | |  |  |  |  |  |  |  |  |  |
| K1 | Would you recommend your GENERAL DOCTOR to a friend or relative? | 0.03 | 0.17 | 0.22 | 0.14 | 0.09 | 0.22 | 0.26 | 0.23 | **0.84** |
| K2 | Would you recommend your GENERAL DOCTOR to someone who does not speak Vietnamese well? | 0.11 | 0.18 | 0.16 | 0.03 | 0.12 | 0.13 | 0.23 | 0.14 | **0.86** |
| K3 | Would you recommend your GENERAL DOCTOR to someone who uses folk medicine, such as herbs or homemade medicines, or has special beliefs about health care? | 0.16 | 0.13 | 0.15 | 0.06 | 0.13 | 0.13 | 0.18 | 0.14 | **0.85** |
